# Pharmacovigilance signal detection of psychiatric adverse events induced by third-generation antiepileptic drugs in children

**DOI:** 10.3389/fphar.2025.1711879

**Published:** 2026-01-05

**Authors:** Wenfang Sun, Yali Li, Binbin Xia, Liushui Li, Jing Chen, Yang Liu, Jingyao Pang, Fang Liu, Hua Cheng

**Affiliations:** Department of Pharmacy, Beijing Luhe Hospital, Capital Medical University, Beijing, China

**Keywords:** children, antiepileptic drugs, FAERS database, psychiatric, adverse event

## Abstract

**Objective:**

The present study was a retrospective pharmacovigilance analysis to identify pharmacovigilance signals of psychiatric adverse events (AEs) associated with the clinical use of third-generation antiepileptic drugs (AEDs) in children, thereby providing a reference for clinical drug selection and pharmaceutical care.

**Methods:**

The data were obtained from the United States Food and Drug Administration (FDA) Adverse Event Reporting System (FAERS). Reports involving patients under 18 years old were retrieved using the generic and brand names of four drugs: lacosamide, perampanel, brivaracetam, and eslicarbazepine. Positive safety signals were detected using the Reporting Odds Ratio (ROR) method and the Bayesian Confidence Propagation Neural Network (BCPNN) method for further analysis. The number of AEs ≥3 cases, ROR value 95% confidence interval lower limit >1 and information component 25 (IC025) must be above 0 was considered statistically significant.

**Results:**

Lacosamide and eslicarbazepine acetate demonstrated relatively lower signals of psychiatric AEs, while brivaracetam was associated with a certain degree of psychiatric risk. In contrast, perampanel requires heightened clinical vigilance due to its potential to induce severe psychiatric and behavioral abnormalities, particularly aggression, suicidal ideation, and homicidal ideation. The most significant psychiatric safety signals for perampanel, based on ROR, were homicidal ideation (ROR = 23.51 [9.72, 56.89]), aggression (ROR = 17.81 [13.85, 22.92]), and psychotic disorders (ROR = 16.94 [9.57, 29.99]).

**Conclusion:**

The findings of this study provide a reference for clinical selection of third-generation AEDs. When initiating treatment in children, clinicians should consider the differential psychiatric risk profiles of these third-generation AEDs, monitor potential psychiatric side effects and adjust prescriptions promptly.

## 1 Introduction

**Epilepsy** is a common neurological disorder, with a prevalence of approximately 0.4%–1.0% in the general population. Children and the elderly represent high-incidence groups, with pediatric prevalence rates reaching up to 5% (Camfield and Camfield, 2015). In children, seizures not only carry the risk of physical injury but may also have long-term adverse effects on cognitive function, language development, motor skills, and social abilities, imposing a substantial burden on families and society (Beghi et al., 2019). At present, pharmacotherapy remains the mainstay of pediatric epilepsy treatment. While antiepileptic drugs (AEDs) can reduce the severity of seizure symptoms, approximately one-third of patients develop treatment resistance (Chen et al., 2018; Steinhoff et al., 2021; Ge et al., 2024). The third-generation AEDs introduced after 2008 have provided new therapeutic options for pediatric epilepsy, featuring unique mechanisms of action and superior efficacy profiles (LaPenna and Tormoehlen, 2017; Roberti et al., 2024).

Evidence suggests that AEDs may increase the risk of adverse events (AEs) in pediatric patients (Toni et al., 2024c). With their increasing clinical use, AEs have become more apparent—particularly psychiatric AEs—which may have profound impacts on mental health and, in some cases, increase the risk of suicide (Strzelczyk and Schubert-Bast, 2022). Existing studies suggest that some third-generation AEDs are associated with psychiatric AEs (Lavu et al., 2022), although the underlying mechanisms remain unclear. However, it is important to note that the psychiatric AEs currently reported for third-generation AEDs are primarily derived from adult studies, with data in the pediatric population remaining limited. Moreover, systematic evaluations of the impact of these drugs on children’s mental health are still scarce.

The United States Food and Drug Administration (FDA) Adverse Event Reporting System (FAERS) is a publicly accessible platform for the spontaneous reporting of adverse drug reactions. With its large volume of relatively standardized reports, FAERS can provide valuable insights into post-marketing drug safety. This study utilizes the FAERS database to perform data mining on AEs associated with four third-generation AEDs—lacosamide, perampanel, brivaracetam, and eslicarbazepine—with a focus on psychiatric AEs, aiming to provide scientific evidence to support clinical drug selection and pharmaceutical monitoring.

## 2 Materials and methods

### 2.1 Data sources

The data for this study were obtained from the official U.S. FDA website (https://fis.fda.gov/extensions/FPD-QDE-FAERS/FPDQDE-FAERS.html). Original ASCII data files from the first quarter of 2004 to the first quarter of 2025 were downloaded and imported into SAS version 9.4 (SAS 9.4) for data cleaning and statistical analysis.

### 2.2 Data processing and extraction

As FAERS data are based on spontaneous reporting, the database contains duplicate or deleted reports. In this study, the unique PRIMARYID from the demographic file (DEMO) was used to link seven data tables—DRUG, REAC, OUTC, RPSR, THER, INDI, and DEMO—according to the FDA’s official data processing guidelines. Reports involving patients under 18 years of age were extracted, and the generic and brand names of the four target AEDs (lacosamide, perampanel, brivaracetam, and eslicarbazepine) were used to identify cases in which the drug was listed as the primary suspect (PS). The latest version of the Medical Dictionary for Regulatory Activities dictionary (MedDRA) v28.0 was applied to re-map and standardize the System Organ Class (SOC) and Preferred Term (PT) designations for AEs in FAERS. The psychiatric PTs included in this study were identified using the SOC code for psychiatric disorders (Code = 10037175).

### 2.3 Data analysis and signal detection

Risk signal detection was performed using the Reporting Odds Ratio (ROR) method and the Bayesian Confidence Propagation Neural Network (BCPNN) method, both of which are based on disproportionality analysis using a 2 × 2 contingency table (Table 1). The ROR method was selected for its computational simplicity and high sensitivity, which minimizes bias from control group selection, albeit at the cost of lower specificity and a potential for false positives. In contrast, the BCPNN method, grounded in Bayesian statistics and a neural network architecture, yields more stable results with higher specificity. To mitigate bias inherent to any single algorithm, we employed a combined approach to leverage the complementary strengths of both methods. The calculation formulas are shown in Table 2.

**TABLE 1:** Two-by-two contingency table for disproportionality analysis.

	Target adverse reactions	Other adverse reactions	Total
	Number of reports	Number of reports	
Target drug	a	b	a+b
Other drug	c	d	c + d
Total	a+c	b + d	a+b + c + d

**TABLE 2:** The principles of disproportionate measurement and the criteria for signal detection.

Method	Calculation formula	Criteria
ROR	$\text{ROR}=\frac{a\;/\;c}{b\;/\;d}$	a ≥3 95%CI (lower limit) > 1
	$SE\left(lnROR\right)=\sqrt{\frac{1}{a}+\frac{1}{b}+\frac{1}{c}+\frac{1}{d}}$	
	$95\%CI=e^{\ln\left(ROR\right)\pm 1.96se}$	
	$\chi 2=\frac{\;{\left(\text{ad}-\text{bc}\right)}^{2}\left(a+b+c+d\right)}{\left(\;a+b\right)\left(a+c\right)\left(c+d\right)\left(b+d\right)}$	
BCPNN	IC = $\log_{2}\frac{p\left(x,y\right)}{p\left(x\right)p\left(y\right)}=\log_{2}\frac{a\left(a+b+c+d\right)}{\left(a+b\right)\left(a+c\right)}$	α1=β1=1; α=β=2; γ11=1 IC025 > 0
	E (IC) = $\log_{2}\frac{\left(a+\gamma 11\right)\left(a+b+c+d+\alpha \right)\left(a+b+c+d+\beta \right)}{\left(a+b+c+d+\gamma \right)\left(a+b+\alpha 1\right)\left(a+c+\beta 1\right)}$	
	$V\left(\text{IC}\right)=\frac{1}{{\left(\ln2\right)}^{2}}\left\{\left[\frac{\left(a+b+c+d\right)-a+\gamma -\gamma 11}{\left(a+\gamma 11\right)\left(1+a+b+c+d+\gamma \right)}\right]+\left[\frac{\left(a+b+c+d\right)-\left(a+b\right)+\alpha -\alpha 1}{\left(a+b+\alpha 1\right)\left(1+a+b+c+d+\alpha \right)}\right]+\left[\frac{\left(a+b+c+d\right)-\left(a+c\right)+\beta -\beta 1}{\left(a+c+\beta 1\right)\left(1+a+b+c+d+\beta \right)}\right]\right\}$	
	$\gamma =\gamma 11\frac{\left(a+b+c+d+\alpha \right)\left(a+b+c+d+\beta \right)}{\left(a+b+\alpha 1\right)\left(a+c+\beta 1\right)}$	
	IC-2SD = E (IC)-2 $\sqrt{V\left(\text{IC}\right)}$	

ROR: reporting odd ratio; BCPNN: bayesian confidence propagation neural network.

In the ROR method, a risk signal was considered present if the number of AE reports (a) was ≥3 and the lower limit of the 95% confidence interval (CI) of the ROR exceeded 1. In the BCPNN method, a signal was identified when the lower bound of the Information Component minus two standard deviations (IC–2SD) was greater than 0 (IC: Information Component; SD: Standard Deviation). Higher values of ROR and IC–2SD indicate stronger associations between the target drug and the suspected AE. In this study, an AE signal was defined as one meeting the threshold criteria in both the ROR and BCPNN analyses. The FAERS provides valuable pharmacovigilance data; however, due to limitations including under-reporting, reporting biases, and lack of denominator data, its findings are inherently hypothesis-generating and cannot establish causality.

### 2.4 Statistical analysis methods

The study performed a descriptive analysis of the four antiepileptic drugs, detailed in tables. This analysis encompassed reporting countries, patient sex and age distribution, reporter profiles, serious AE, and patient outcomes. All statistical analyses and data mining were performed using SAS 9.4. Categorical data are presented as proportions (%). Disproportionality analyses for psychiatric AEs utilized the ROR and IC_025_.

## 3 Results

### 3.1 Basic information on AE reports

A total of **23,554** AE reports were extracted for the present study, of which **1,854** were pediatric reports, accounting for **7.87%** of the total. Among these, lacosamide accounted for **1,154** reports, perampanel for **379**, brivaracetam for **213**, and eslicarbazepine for **108**. Reports involving female pediatric patients numbered **850** (**45.85%**), male pediatric patients **903** (**48.71%**), and cases with unknown gender **101** (**5.45%**). The mean age at drug administration was **9.38 years**. The majority of AE reports were submitted by physicians or through spontaneous patient self-reporting. Severe AEs accounted for 78.59% of all reports, with perampanel showing the highest proportion of severe AEs (90.77% of its total reports) and the highest mortality rate (4.75%). Lacosamide was associated with the greatest number of deaths (52 cases), exceeding those reported for the other three drugs. Basic demographic and reporting characteristics are summarized in Table 3.

**TABLE 3:** Basic information on adverse event reports for four antiepileptic drugs in FAERS.

Item	Group	Lacosamide n = 1154 (%)	Perampanel n = 379 (%)	Brivaracetam n = 213 (%)	Eslicarbazepine n = 108 (%)	Total n = 1854 (%)
Gender	Female	530 (45.93)	176 (46.44)	85 (39.91)	59 (54.63)	850 (45.85)
	Male	552 (47.83)	192 (50.66)	112 (52.58)	47 (43.52)	903 (48.71)
	Unknown	72 (6.24)	11 (2.90)	16 (7.51)	2 (1.85)	101 (5.45)
Age	<1year	123 (10.66)	12 (3.17)	15 (7.04)	2 (1.85)	152 (8.20)
	1–5years	195 (16.90)	43 (11.35)	24 (11.27)	20 (18.52)	282 (15.21)
	5–12 years	428 (37.09)	110 (29.02)	68 (31.92)	46 (42.59)	652 (35.17)
	12–17 years	408 (35.36)	214 (56.46)	106 (49.77)	40 (37.04)	768 (41.42)
	Mean (SD)	8.66 (5.47)	10.95 (4.98)	10.33 (5.31)	9.69 (5.06)	9.38 (5.41)
Reporter	Pharmacist	194 (16.81)	42 (11.08)	18 (8.45)	9 (8.33)	263 (14.19)
	Physician	480 (41.59)	280 (73.88)	79 (37.09)	36 (33.33)	875 (47.20)
	Patient	403 (34.92)	23 (6.07)	113 (53.05)	52 (48.15)	591 (31.88)
	Other	63 (5.46)	28 (7.39)	3 (1.41)	10 (9.26)	104 (5.61)
	Unknown	14 (1.21)	6 (1.58)	0 (0.00)	1 (0.93)	21 (1.13)
Report severity	Serious reports	882 (76.43)	344 (90.77)	168 (78.87)	63 (58.33)	1457 (78.59)
	Non-serious reports	272 (23.57)	35 (9.23)	45 (21.13)	45 (41.67)	397 (21.41)
Types of serious reports	Life threatening	33 (2.86)	27 (7.12)	3 (1.41)	0 (0.00)	63 (3.4)
	Hospitalization	378 (32.76)	187 (49.34)	44 (20.66)	18 (16.67)	627 (33.83)
	Disability	8 (0.69)	11 (2.90)	2 (0.94)	0 (0.00)	21 (1.13)
	Death	52 (4.51)	18 (4.75)	9 (4.23)	1 (0.93)	80 (4.31)
	Congenital malformation	29 (2.51)	4 (1.06)	2 (0.94)	2 (1.85)	37 (2.00)
	Other	382 (33.10)	97 (25.59)	108 (50.70)	42 (38.89)	629 (33.93)
Year of first Reported case		2009	2013	2016	2014	

### 3.2 SOC-level analysis of detected signals

Positive signals for the four AEDs involved a total of **22** SOCs, with lacosamide involving **16** SOCs, perampanel **12**, brivaracetam **10**, and eslicarbazepine **7**. SOCs common to all four drugs included: nervous system disorders, general disorders and administration site conditions, psychiatric disorders, investigations (clinical and laboratory assessments). The highest number of positive signals was detected for **lacosamide** (**1,669 cases**), followed by perampanel (**541**), brivaracetam (**364**), and eslicarbazepine (**129**). The number of PTs involved was **83** for lacosamide, **51** for perampanel, **30** for brivaracetam, and **14** for eslicarbazepine. The SOC distribution of AEs for the four drugs is shown in Table 4.

**TABLE 4:** SOC distribution of AEs associated with four antiepileptic drugs.

SOC	Lacosamide (n)		Perampanel (n)		Brivaracetam (n)		Eslicarbazepine (n)	
	AE	PT	AE	PT	AE	PT	AE	PT
Nervous system disorders	709	30	195	17	144	9	63	4
General disorders and administration site conditions	272	9	47	6	24	3	29	2
**Psychiatric disorders**	**27**	**3**	**178**	**14**	**48**	**7**	**13**	**3**
Investigations	25	4	6	1	10	1	4	1
Injury, poisoning and procedural complications	416	9	58	5	96	4		
Gastrointestinal disorders	8	2	8	1			4	1
Infections and infestations	40	3	24	2				
Metabolism and nutrition disorders	26	4			8	1	3	1
Skin and subcutaneous tissue disorders			5	1			13	2
Product issues	7	1			14	2		
Hepatobiliary disorders	8	2						
Neoplasms benign, malignant and unspecified (incl cysts and polyps)	3	1						
Surgical and medical procedures	21	3			13	1		
Pregnancy, puerperium and perinatal conditions	8	1			3	1		
Renal and urinary disorders			5	1				
Musculoskeletal and connective tissue disorders			5	1				
Cardiac disorders	72	8						
Eye disorders	16	2						
Congenital, familial and genetic disorders	11	1						
Respiratory, thoracic and mediastinal disorders			7	1				
Ear and labyrinth disorders			3	1				
Social circumstances					4	1		
Total	1669	83	541	51	364	30	129	14

SOC: system organ class; AE: adverse event; PT: preferred term. The bold values: Focus on psychiatric AEs associated with the four drugs.

### 3.3 Detection of psychiatric disorder–related signals

The psychiatric disorder–related PTs for the four drugs are summarized in Table 5.

- **Perampanel** was associated with **14** psychiatric PTs, accounting for **178 AE reports**, making it the drug with the highest number of psychiatric AEs among the four. Frequently reported PTs included aggression (**65 cases**), suicidal ideation (**18 cases**), agitation (**16 cases**), suicide attempt (**15 cases**), and irritability (**15 cases**). Strongly associated PTs included homicidal ideation (**ROR = 23.51 [9.72, 56.89]**), aggression (**ROR = 17.81 [13.85, 22.92]**), psychotic disorder (**ROR = 16.94 [9.57, 29.99]**), self‐injurious behaviour (**ROR = 10.45 [3.35, 32.55]**), and affect lability (**ROR = 8.53 [2.74, 26.55]**).
- **Brivaracetam** had **48** psychiatric AE reports. The three most common PTs were aggression (**19 cases**), insomnia (**7 cases**), and suicidal ideation (**6 cases**). The PTs with the strongest associations were behaviour disorder (**ROR = 18.01 [7.45, 43.51]**), nervousness (**ROR = 8.67 [2.79, 27.00]**), and psychotic disorder (**ROR = 8.17 [3.05, 21.85]**).
- **Lacosamide** was associated with **3** psychiatric PTs: irritability (**17 cases**, **ROR = 2.04 [1.27, 3.29]**), behaviour disorder (**6 cases**, **ROR = 4.69 [2.10, 10.47]**), and staring (**4 cases**, **ROR = 5.78 [2.16, 15.49]**).
- **Eslicarbazepine** had the fewest psychiatric AEs, including suicidal ideation (**5 cases**, **ROR = 7.01 [2.89, 16.98]**), irritability (**4 cases**, **ROR = 5.80 [2.16, 15.56]**), and abnormal behavior (**4 cases**, **ROR = 4.21 [1.57, 11.30]**).

**TABLE 5:** Psychiatric disorder–related Preferred Terms and associated ROR values for four antiepileptic drugs.

Symptom domain	PT (preferred term)	Perampanel N = 541			Lacosamide N = 1669			Brivaracetam N = 364			Eslicarbazepine N = 129		
		ROR(95%CI)	IC(IC025)	n (%)	ROR(95%CI)	IC(IC025)	n (%)	ROR(95%CI)	IC(IC025)	n (%)	ROR(95%CI)	IC(IC025)	n (%)
Mood disorders	**Irritability**	**5.71(3.42,9.51)**	**2.49(1.40)**	**15(2.77)**	**2.04(1.27,3.29)**	**1.02(0.26)**	**17(1.02)**				**5.80(2.16,15.56)**	**2.52(0.25)**	**4(3.10)**
	Agitation	5.37 (3.27,8.80)	2.40 (1.37)	16 (2.96)									
	Restlessness	5.85 (2.62,13.06)	2.54 (0.69)	6 (1.11)									
	Affect lability	8.53 (2.74,26.55)	3.08 (0.11)	3 (0.55)									
	Depressed mood	4.90 (2.04,11.82)	2.29 (0.38)	5 (0.92)									
	Nervousness							8.67 (2.79,27.00)	3.11 (0.12)	3 (0.82)			
	Mood swings							4.90 (1.83,13.11)	2.29 (0.16)	4 (1.10)			
Behavioral disturbance	**Behaviour disorder**	**7.33(2.36,22.83)**	**2.87(0.05)**	**3(0.55)**	**4.69(2.10,10.47)**	**2.22(0.51)**	**6(0.36)**	**18.01(7.45,43.51)**	**4.15(1.04)**	**5(1.37)**			
	**Aggression**	**17.81(13.85,22.92)**	**4.06(3.38)**	**65(12.01)**				**7.30(4.62,11.52)**	**2.83(1.79)**	**19(5.22)**			
	**Suicidal ideation**	**6.62(4.15,10.56)**	**2.70(1.66)**	**18(3.33)**				**3.19(1.43,7.13)**	**1.67(0.18)**	**6(1.65)**	**7.01(2.89,16.98)**	**2.78(0.60)**	**5(3.88)**
	Suicide attempt	5.30 (3.18,8.82)	2.39 (1.32)	15 (2.77)									
	Homicidal ideation	23.51 (9.72,56.89)	4.53 (1.11)	5 (0.92)									
	Self-injurious behaviour	10.45 (3.35,32.55)	3.37 (0.18)	3 (0.55)									
	Anger	4.66 (2.32,9.35)	2.21 (0.75)	8 (1.48)									
	Staring				5.78 (2.16,15.49)	2.52 (0.26)	4 (0.24)						
	Insomnia							3.37 (1.60,7.11)	1.74 (0.34)	7 (1.92)			
	Abnormal behaviour										4.21 (1.57,11.30)	2.06 (0.04)	4 (3.10)
Psychosis	**Psychotic disorder**	**16.94(9.57,29.99)**	**4.06(2.11)**	**12(2.22)**				**8.17(3.05,21.85)**	**3.02(0.45)**	**4(1.10)**			
	Hallucination, visual	6.14 (2.30,16.42)	2.61 (0.30)	4 (0.74)									
Total				178 (32.90)			27 (1.62)			48 (13.19)			13 (10.08)

PT: preferred term; ROR: reporting odd ratio; IC: information components; The bold values: PT present in two or more drugs.

### 3.4 Shared and drug-specific associations of psychiatric AEs

Among the 19 psychiatric AEs identified across the four drugs, three were concurrently associated with three different agents. When ranked by the strength of the ROR, the signal for suicidal ideation was strongest for eslicarbazepine, followed by perampanel and then brivaracetam. Similarly, the signal for irritability was most pronounced for eslicarbazepine, then perampanel, and finally lacosamide. The signal for behavior disorder was strongest for brivaracetam, followed by perampanel and lacosamide. Both perampanel and brivaracetam were associated with aggression and psychotic disorder. However, the strength of the signals for these AEs was markedly higher with perampanel. Several signals were uniquely identified in individual drugs: nervousness, insomnia, and mood swings were found only for brivaracetam; staring was specific to lacosamide; and abnormal behavior was a distinctive signal for eslicarbazepam. Lastly, a cluster of signals—including agitation, suicide attempt, anger, restlessness, homicidal ideation, depressed mood, Hallucination visual, affective lability and self-injurious behaviour—was exclusively associated with perampanel.

## 4 Discussion

The four third-generation AEDs included in this study—lacosamide, perampanel, brivaracetam, and eslicarbazepine—were reported to cause AEs in children beginning in 2009, 2013, 2016, and 2014, respectively. All four drugs have been approved by the FDA for the monotherapy or adjunctive treatment of partial-onset seizures in children aged 4 years and older. In this study, lacosamide accounted for the highest proportion of AE reports (62.24%), which may be related to its earlier market approval. The majority of drug administrations occurred in children aged over 8 years, with a mean age of 9.38 years. However, AEs were also reported in children under 4 years old, indicating off-label use of all four drugs. This finding highlights the need for clinicians to pay close attention to age-related prescribing restrictions.

Analysis of SOC involvement revealed that lacosamide-related AEs were primarily concentrated in nervous system disorders, injury/poisoning/procedural complications, and general disorders and administration site conditions. This study also identified SOC categories for lacosamide that were not listed as key concerns in its prescribing information, including infections and infestations, as well as metabolic and nutritional disorders. Both perampanel and brivaracetam were mainly associated with nervous system disorders, psychiatric disorders, and injury/poisoning/procedural complications. Eslicarbazepine-related AEs were primarily concentrated in nervous system disorders, general disorders and administration site conditions, psychiatric disorders, and skin/subcutaneous tissue disorders. Notably, psychiatric disorders SOCs were implicated for all four drugs.

Perampanel is currently the only marketed highly selective, non-competitive α-amino-3-hydroxy-5-methyl-4-isoxazolepropionic acid (AMPA)–type glutamate receptor antagonist (Maguire et al., 2022). In this study, perampanel was associated with the most extensive profile of psychiatric AEs, which constituted 32.90% of all its reported AEs. Upon its market approval, the U.S. FDA issued a boxed warning stating that the drug may cause severe, potentially life-threatening psychiatric and behavioral changes, including aggression, agitation, anger, and homicidal or suicidal ideation (Frampton, 2015; Liu et al., 2023b). This study confirmed the presence of these signals in the pediatric population, with significant RORs, such as aggression (**ROR = 17.81 [13.85, 22.92]**), agitation (**ROR = 5.37 [3.27, 8.80]**), suicidal ideation (**ROR = 6.62 [4.15, 10.56]**). Due to its psychiatric risk profile, perampanel was classified as a Schedule II psychotropic drug in China as of 1 July 2023. In this study, perampanel was found to be associated with the highest number of suicide-related signals, including suicidal ideation, suicide attempts, homicidal ideation and self-injurious behaviour. This finding is consistent with previous research (Leppien et al., 2023). The mechanism underlying its psychiatric adverse effects remains unclear, but may involve serotonin, γ-aminobutyric acid (GABA), and glutamate pathways (Moraes et al., 2020).

Lacosamide is a voltage-gated sodium channel blocker that exerts its antiepileptic effect through selective binding to the slow inactivation state of Na^+^ channels (Cho et al., 2022). In the present study, lacosamide had the earliest market approval and the highest total number of AE reports among the four drugs, but the lowest proportion of psychiatric AEs (1.62%). These were mainly conduct disorder, irritability, and staring—AEs not listed in the prescribing information. Some studies have suggested that lacosamide may even improve cognitive and behavioral outcomes in epilepsy patients (Liguori et al., 2018), which may explain its relatively mild psychiatric impact. Despite evidence of suicide-related signals for lacosamide in adults (Liu et al., 2023a), our study found no such association in children.

Eslicarbazepine is a derivative of carbamazepine that reduces neuronal excitability by selectively inhibiting voltage-gated sodium channels on neuronal membranes, thereby decreasing sodium influx and suppressing seizure activity (Leslie et al., 2020). In this study, eslicarbazepine had the fewest total AEs, likely due to its later market introduction. Psychiatric AEs accounted for **10.08%** of its reports, mainly irritability, suicidal ideation, and abnormal behavior. A multinational, parallel-group study reported that eslicarbazepine effectively controlled seizures in children with no serious cognitive or neuropsychological adverse effects and was well tolerated in long-term use (Veggiotti et al., 2022), findings that are consistent with our results. Some studies indicate that irritability and abnormal behavior are more frequently observed in children than in adults (Tang et al., 2024). This heightened susceptibility in children may be attributed to increased sensitivity of the developing central nervous system to eslicarbazepine, potentially leading to a spectrum of more complex and overt behavioral manifestations.

Brivaracetam, a structural derivative of levetiracetam, is a highly selective ligand for synaptic vesicle protein 2A (SV2A) on presynaptic neuronal membranes, exerting its antiepileptic action by modulating synaptic function (Tulli et al., 2021). In the present study, brivaracetam ranked second to perampanel in the proportion of psychiatric AEs (**13.19%**), most commonly aggression, insomnia, and suicidal ideation. In adults, the most common psychiatric AEs associated with brivaracetam were aggression, suicidal ideation, and behaviour disorder (Meng et al., 2025). While this spectrum of AEs was largely consistent with that observed in children, the strength of the drug-event association was notably higher in adults. Studies show that these psychiatric AEs are mostly mild to moderate in severity and rarely necessitate discontinuation of therapy (Steinhoff et al., 2021).

In the present study, the incidence of psychiatric AEs was highest with perampanel, followed by brivaracetam, eslicarbazepine, and lacosamide. Many of these psychiatric AEs are insidious and non-specific in onset, such as mood changes and insomnia, which can easily be misattributed to the underlying epilepsy, comorbid conditions, or normal childhood behavioral variations (Hermann et al., 2017). Prior to initiating any third-generation AED therapy, it is recommended to establish a baseline assessment of the child’s mental status and implement a protocol for proactive, structured monitoring. This should involve the regular use of brief psychiatric and behavioral rating scales, particularly during dose titration and maintenance phases, to systematically query both the child and caregivers about changes in mood, sleep, behavior, and social engagement (Fecske et al., 2020; Ren et al., 2023). Upon identification of a suspected and clinically relevant psychiatric AE, clinicians should first evaluate its relationship with the underlying disease or comorbidities. Subsequently, depending on the event’s severity, management strategies may include dose reduction, switching to an alternative agent with a more favorable psychiatric side effect profile (such as lacosamide), or discontinuation of the medication if necessary (Liu C. et al., 2025). Early screening and timely intervention are crucial measures for preventing the development of refractory epilepsy (Guilfoyle et al., 2015; Hoxhaj et al., 2023).

In this study, aggression was the most frequent psychiatric AE associated with perampanel and brivaracetam, with RORs of 17.81 (13.85, 22.92) and 7.30 (4.62, 11.52), respectively. It is imperative that clinicians acknowledge the substantial risk of aggression, suicidal ideation, and other psychiatric AEs associated with perampanel and brivaracetam in children. This risk mandates proactive identification of high-risk patients, including those with pre-existing psychiatric conditions, intellectual disability, or behavioral disorders (Liu Y.-H. et al., 2025), who require intensified monitoring and pre-emptive counseling. Evidence suggests that discontinuation of perampanel can reduce or even completely resolve aggressive behaviors, whereas in patients who continue treatment, dose reduction can decrease the occurrence of such behaviors (Mammì et al., 2022). One study found that AEs associated with perampanel were dose-dependent, with higher doses (12 mg) posing a greater risk of psychiatric side effects such as irritability, aggression, and anger (Fan et al., 2023), without providing significantly greater therapeutic benefits compared to lower doses (8 mg) (Bresnahan et al., 2023). The management of perampanel should follow a “start-low, go-slow” titration strategy due to its dose-related neuropsychiatric risks (Huang et al., 2022), with careful consideration of higher doses to optimize the risk-benefit ratio.

Pharmacovigilance in high-risk pediatric populations, such as those with chronic conditions including congenital heart disease, epilepsy, and rare diseases (De Oliveira et al., 2023; Toni et al., 2024a), presents significantly greater complexity compared to that in general adult or even the general pediatric population. This increased complexity stems primarily from their unique physiological characteristics and distinct metabolic responses to medications (Elzagallaai et al., 2017). Many chronic conditions necessitate pharmacotherapy for years or even a lifetime. However, the long-term cumulative toxicities of medications that appear safe in the short term—such as their effects on neurodevelopment, growth, and organ function—cannot be reliably detected in pre-marketing studies due to their limited duration (Padhi et al., 2024). The real-world experience with some third-generation anti-epileptic drugs in pediatric populations remains limited. Our study, by leveraging post-marketing big data for signal detection, aims to address this critical evidence gap left by the inherent limitations of pre-marketing clinical trials. The risk signals identified in our study (e.g., perampanel with aggression) establish a foundational feature set for future predictive machine learning models (Toni et al., 2024b). Such models could integrate genotype, underlying disease, comorbid psychiatric history, concomitant medications, and pharmacokinetic parameters to generate individualized risk profiles for severe psychiatric AEs before treatment initiation, enabling pre-emptive management and refined monitoring strategies (Liu et al., 2012). The implementation of these targeted monitoring and intervention strategies will facilitate the timely detection and management of drug-induced psychiatric AEs.

This study has several limitations. First, the FAERS database is a spontaneous reporting system, and the variability in reporters’ expertise and standards may affect data quality, potentially introducing bias. Second, the study cannot establish a causal relationship between drug use and AEs, nor can it determine the incidence of specific reactions; further clinical trials are needed for confirmation. Third, epilepsy itself can cause mental health problems and is frequently comorbid with various psychiatric disorders (Holmes, 2021). Patients with epilepsy often have lower mental health scores compared to those without epilepsy, making it difficult to ascertain whether psychiatric adverse effects are drug-induced or a consequence of exacerbated underlying psychiatric conditions. Fourth, third-generation AEDs offer advantages such as fewer drug–drug interactions and lower rates of adverse reactions, making them highly promising for pediatric epilepsy treatment; however, their safety—particularly regarding cognitive impact—still requires validation through large-scale, long-term clinical studies.

## 5 Conclusion

This study systematically analysed the psychiatric AEs associated with four third-generation AEDs in children, based on data mining of the FAERS database. This study provides important data supporting the psychiatric safety profiles of these AEDs in children and underscores the necessity of monitoring their potential psychological impacts in clinical practice. Future high-quality prospective studies are warranted to establish more reliable evidence for the rational use of these medications and mental health management in pediatric epilepsy patients.

## Data Availability

The datasets presented in this study can be found in online repositories. The names of the repository/repositories and accession number(s) can be found in the article/supplementary material.
